# Improving Family Medicine Residency Training in Women’s Health-Related Procedures at a Community Hospital

**DOI:** 10.7759/cureus.85181

**Published:** 2025-06-01

**Authors:** Erum Azhar, Sobia Zareen, Hira Fatima, Mudasir Umer, Francis Guerra-Bauman, Syed Atif, Trajan Barrera, Feroza Patel, Saniya Kamal, Muhammad Sheraz Yousaf, Abdul Waheed

**Affiliations:** 1 Obstetrics and Gynecology, Dignity Health East Valley, Gilbert, USA; 2 Obstetrics and Gynecology, Creighton University School of Medicine, Phoenix, USA; 3 Family and Community Medicine, Baylor College of Medicine, Houston, USA; 4 Family Medicine, WellSpan Good Samaritan Hospital, Lebanon, USA; 5 Family Medicine, International University of Kyrgyzstan, Bishkek, KGZ; 6 Family Medicine, Fairfield Memorial Hospital, Fairfield, USA; 7 Internal Medicine, Medstar Health, Baltimore, USA; 8 Family and Community Medicine, Creighton University School of Medicine, Phoenix, USA; 9 Family Medicine, Dignity Health Medical Group, Gilbert, USA

**Keywords:** accreditation council for graduate medical education (acgme), continuous process improvement, family medicine residency program, procedural skills training, quality improvement projects

## Abstract

Introduction: The Accreditation Council for Graduate Medical Education requires family medicine (FM) residents to be trained in outpatient procedures. The Society of Teachers of Family Medicine (STFM) group on hospital and procedural training compiled a core list of procedures that all FM residents should perform by graduation. We implemented a quality improvement project using a bundled intervention to enhance resident training in outpatient gynecological procedures.

Materials and methods: This quasi-experimental study evaluated a multifaceted bundled intervention. Baseline data were collected from January 2018 to June 2019, with intervention phases occurring from June 2019 to February 2020 and March 2021 to November 2022. Stakeholders collaborated to identify barriers using process mapping and Ishikawa diagrams. The JMP Pro 17 software (JMP Statistical Discovery LLC, Cary, NC, USA) was used to create an X-mR statistical process control (SPC) chart for phase analysis. Poisson regression analysis was conducted to assess the impact of the intervention on the number of women’s health-related procedures performed per resident per month. Residents’ graduation portfolios were reviewed for documentation of procedural competency.

Results: The intervention increased the average number of procedures from two to six per resident per month. Significant increases in procedural volume were observed during phase 1 (roll-out) and phase 3 (post-intervention), with a temporary stall in phase 2 due to COVID-19 disruptions. Poisson regression analysis showed statistically significant increases in incidence rate ratios during phases 1 and 3 (p<0.005). Documentation of competency in women’s health-related procedures at graduation also improved over time.

Conclusions: An FM residency training program successfully utilized a multifaceted, bundled intervention to increase the number of gynecological procedures performed at FM practice sites, aligning resident training with STFM recommendations.

## Introduction

Training in outpatient women’s health-related procedures is a critical component of family medicine (FM) residency programs, ensuring that FM physicians are equipped to provide comprehensive women’s healthcare. According to the Accreditation Council for Graduate Medical Education (ACGME), FM residents must demonstrate proficiency in these procedures as part of their training requirements [1]. Similarly, the Society of Teachers of Family Medicine (STFM) has identified key women’s health-related procedures, such as intrauterine device (IUD) insertion, vulvar biopsy, endometrial biopsy, and cervical polyp removal, as essential for FM residents’ training [2].

Despite their importance, many academic generalists infrequently perform women’s health-related procedures, leading to low confidence in precepting these skills [3]. This issue is particularly significant in rural and underserved areas, where FM physicians often serve as the primary providers of women’s healthcare. While specialized obstetricians and gynecologists are present in only 60% of U.S. counties, FM physicians are available in over 90%, making them a vital resource for addressing women’s healthcare needs, including contraception and pregnancy-related care [4,5].

However, confidence among FM physicians in performing women’s health-related procedures remains low [2]. Studies show that limited exposure during residency contributes to reduced procedural competence and a reluctance to offer these services in practice [3]. For example, fewer than 20% of FM physicians perform IUD insertions, with even fewer offering implants or other advanced women’s health-related procedures [6,7].

This training gap has significant implications for patient care, ranging from reducing unintended pregnancies to early detection of malignancies. It also impacts healthcare costs by decreasing referrals and hospitalizations [5,8].

To address these gaps, this study implemented a quality improvement project aimed at enhancing the training of FM residents in outpatient women’s health-related procedures through a multifaceted, bundled intervention. This included preceptor training, the creation of a dedicated women’s health-related procedure clinic, didactic sessions, and regular simulation training. This approach aims to improve training capacity, provide preliminary data to inform future training protocols, and ensure that FM physicians are well-prepared to meet the growing demand for comprehensive women’s healthcare services.

## Materials and methods

Study design and context

**Figure 1:**
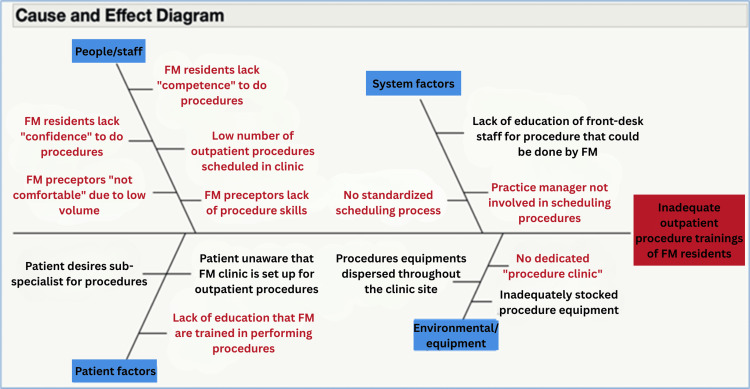
Ishikawa/fishbone diagram indicating the factors associated with inadequate women’s health-related procedures FM: family medicine

**Figure 2:**
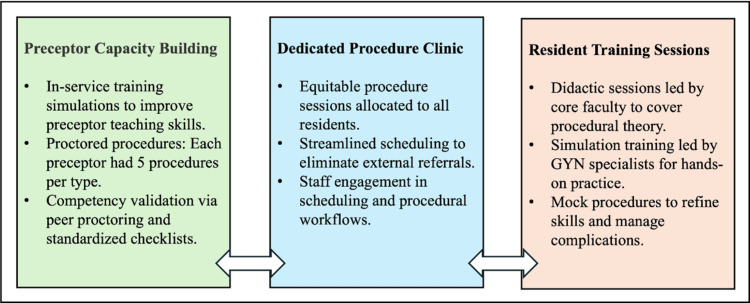
Schematic indicating the major building blocks of the multifaceted intervention bundle GYN: gynecology

This quasi-experimental study implemented a multifaceted bundled intervention. Baseline data were collected from January 2018 to June 2019. Stakeholders, including clinic staff, practice managers, residents, program leadership, and faculty, conducted process mapping and Fishbone/Ishikawa diagram exercises (Figure 1) to identify factors contributing to low women’s health-related procedure rates. Using the Pareto principle (also known as the 80/20 rule), an intervention bundle was developed (Figure 2) and implemented from June 2019 to February 2020. Due to COVID-19 shutdowns, some components, including a dedicated procedure clinic, were paused and later fully reinstated from March 2021 to November 2022.

The main elements of the intervention bundle are shown in Figure 2. This bundle resulted from the utilization of modern quality improvement tools, including stakeholder engagement, a pre-intervention process map, and an Ishikawa diagram (Figure 1). Figure 2 illustrates that the intervention bundle focused on provider-facing efforts, such as capacity building among faculty preceptors and resident training, as well as operations-facing efforts, including process improvements in scheduling through a dedicated procedure clinic. Additionally, it involved patient-facing efforts, including education on the procedures performed in the clinic. Resident competency in individual procedures was assessed by preceptors using a standardized form electronically within the Residency Management Suite (New Innovations, Inc., Uniontown, OH, USA), which is used by our program.

Using the Pareto principle (also known as the 80/20 rule), an intervention bundle was developed (Figure 2) and implemented from June 2019 to February 2020. Due to COVID-19 shutdowns, some components, including the dedicated procedure clinic, were temporarily paused and later fully reinstated from March 2021 to November 2022.

Study population

The study was conducted at two FM residency clinics and involved 22 residents, 15 faculty members, and four advanced practice providers. The study was approved by the Institutional Review Board of WellSpan Hospital as an exempt quality improvement project, which does not require full board review.

Data collection

Data were collected monthly using the SlicerDicer logic model in the Epic electronic medical record (Epic Systems Corporation, Verona, WI, USA). All women’s health-related procedures, except for Pap smears and maternity care, were included. Pap smears were excluded because residents had already achieved sufficient numbers and demonstrated competency at the outset of this project. Similarly, maternity care-related procedures were addressed in a separate curriculum and were not a concern within the program. Residents’ competency documentation for all graduates in 2019, 2020, 2021, and 2022 was also reviewed for women’s health-related procedures by examining the final portfolios evaluated by the last Clinical Competency Committee using the Residency Management Suite. This manuscript was prepared following the Standards for Quality Improvement Reporting Excellence (SQUIRE) 2.0 guidelines.

Data analysis

Statistical process control (SPC) charts were used to visualize trends, and the Procedure Quality Improvement Committee reviewed the data. A phase analysis was conducted using the JMP Pro 17 software (JMP Statistical Discovery LLC, Cary, NC, USA) on X-mR SPC charts. The Institute for Healthcare Improvement rules were applied to detect any shifts or drifts in the process on phase analysis displays. Poisson regression analysis with interrupted time series was employed to evaluate the impact of the intervention. A p-value of <0.005 was considered statistically significant.

## Results

**Figure 3:**
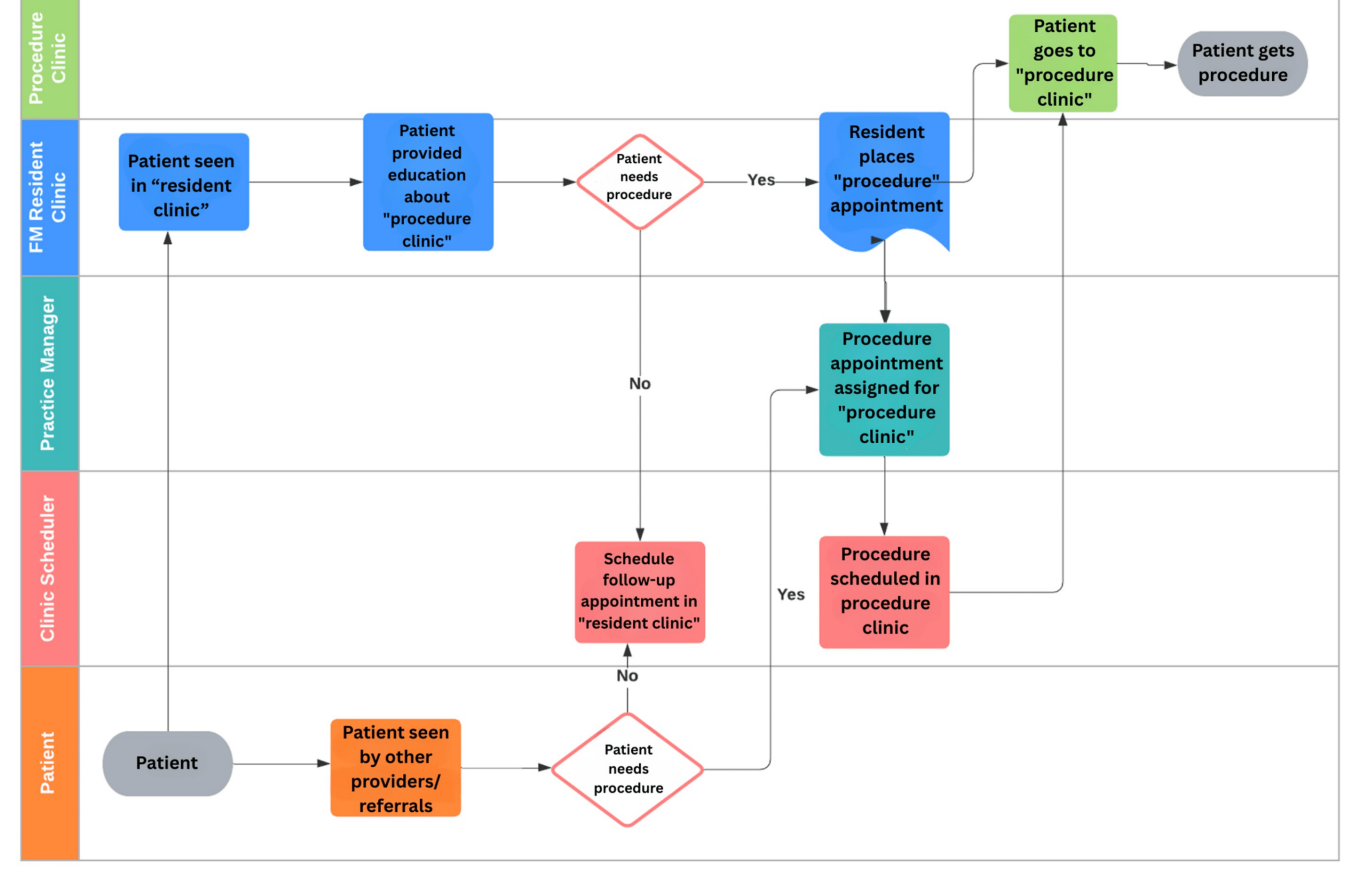
Post-intervention process map with swim lane

The project was successfully conducted through four phases. Figure 3 shows the post-intervention process map. It depicts the swim-lane process leading to scheduling an appointment with a resident in a dedicated procedure clinic, where the procedure was ultimately performed. A noteworthy element in the post-intervention process map is the single outcome at the end, as opposed to the multiple pathways observed pre-intervention.

**Figure 4:**
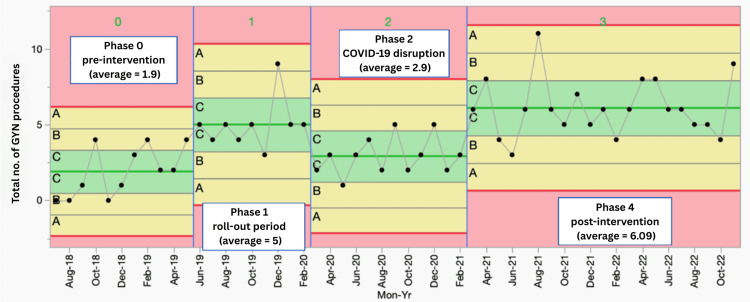
Phase analysis of the data over time using SPC charts An X-mR statistical process control (SPC) chart showing individual values (X) with a moving range (mR) of the number of gynecological (GYN) procedures per resident per month per resident per month from October 2018 to November 2022, segmented into four operational phases with corresponding shifts in control limits and average number of procedures. The vertical blue line shows the transitions between the phases, and μ0 represents the mean as the center green line. The upper red line is the three-sigma upper control limit (UCL). The lower red line is the three-sigma lower control limit (LCL). The UCL and LCL represent three standard deviations above and below the mean, respectively.

Figure 4 shows the four-phase analysis of women’s health-related procedures performed per resident per month on an X-mR SPC chart. An X-mR SPC chart displays individual values (X) with a moving range (mR) of the number of gynecological procedures per resident per month from October 2018 to November 2022. The data are segmented into four operational phases, with corresponding shifts in control limits and the average number of procedures. The vertical blue lines indicate the transitions between phases, and μ₀ represents the mean, shown as the center green line. The upper red line is the three-sigma upper control limit (UCL), while the lower red line is the three-sigma lower control limit (LCL). The UCL and LCL represent three standard deviations above and below the mean, respectively.

The chart shows a clear shift in the process from the baseline (phase 0) during the roll-out period (phase 1). The number of women’s health-related procedures performed per resident per month increased from 1.9 in phase 0 to 5 in phase 1, the roll-out period. This number then dropped from 5 to 2.9 in phase 2, which corresponds to the start of the COVID-19 pandemic. Although the drop in phase 2 indicates a slight drift in the process, this was due to COVID-19 disruptions and the closure of parts of the dedicated women’s health-related procedure clinic. It is noteworthy that the number of procedures performed per resident per month in phase 2 remained higher than in phase 0.

Entering the post-intervention period, phase 3, there is a clear shift in the process, indicating a significant change associated with the full implementation of our multifaceted bundled intervention. In this phase, the number of women’s health-related procedures performed per resident per month increased from 2.9 to 6.09, which is higher than in phases 2, 1, and 0, indicating a significant shift in the process.

**Figure 5:**
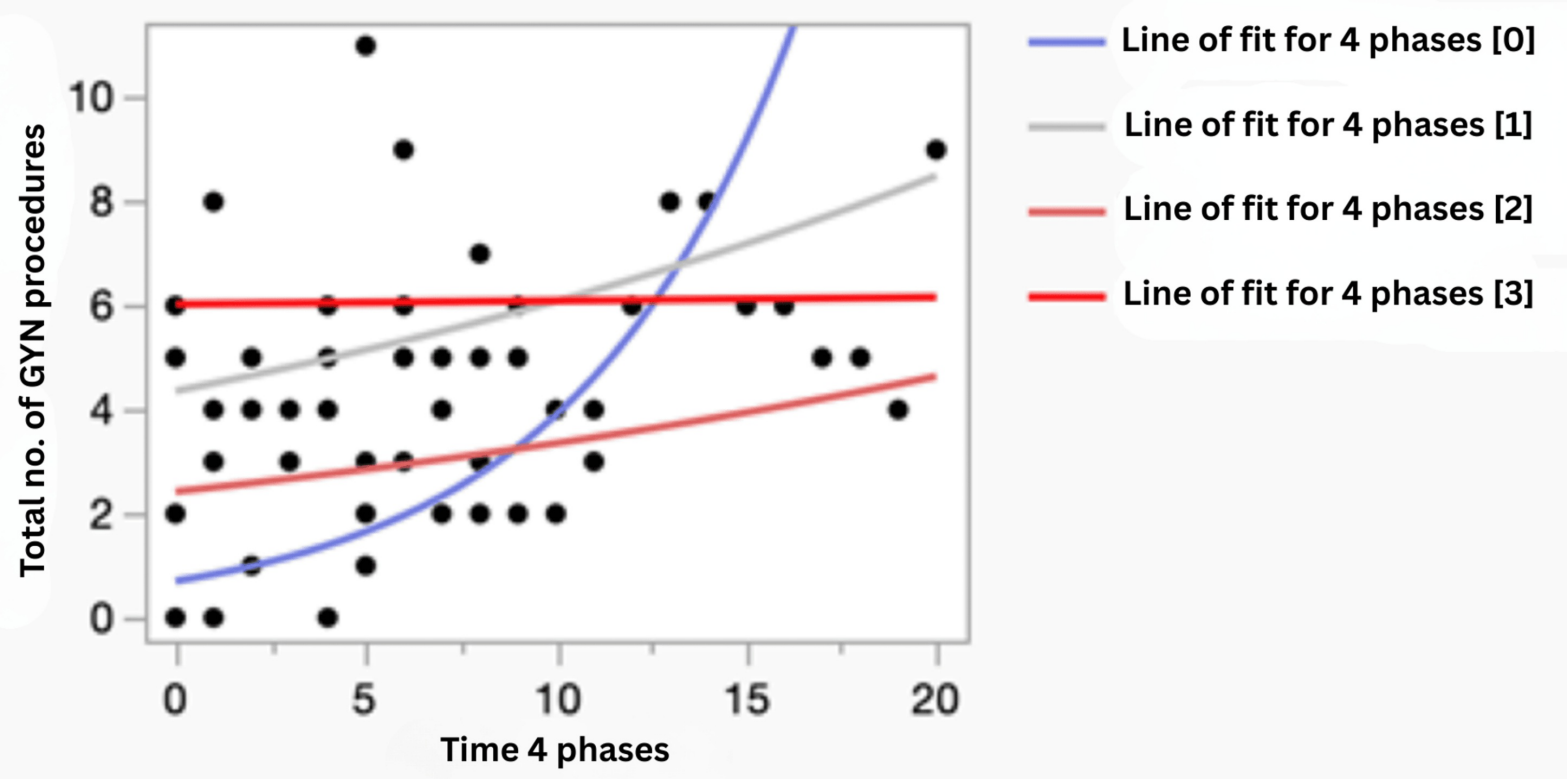
Regression plot of maximum likelihood of total women’s health-related procedures per resident per month on Poisson distribution GYN: gynecological

Poisson regression analysis further confirmed the findings from the phase analysis on the SPC. Figure 5 shows the regression plot of the maximum likelihood of the total number of women’s health-related procedures per resident per month based on a Poisson distribution. The incidence rate ratios, along with their 95% confidence intervals (CI), for the number of procedures per resident per month from baseline to the three other phases are shown in Table 1. Although the number increased from baseline throughout the different phases, it was statistically significant only for phases 1 and 3, which correspond to the direct pre-implementation and post-implementation phases. The only significant difference in the process between these two phases, as compared to phases 1 and 3, was the introduction of a dedicated women’s health-related procedure clinic. This indicates that the implementation of a dedicated procedure clinic with process improvement was associated with a significant increase (p<0.05) in the number of women’s health-related procedures performed per resident per month in our residency program.

**Table 1:** Incidence rate ratios for four phases of the primary outcome of the total number of women’s health-related procedures per resident per month

Level 1	Level 2	Incidence rate ratio	Lower 95% confidence interval	Lower 95% confidence interval
Phase 3 (post-intervention)	Phase 0 (pre-intervention)	2.65	1.65	4.24
Phase 2 (COVID-19 disruption)	Phase 0 (pre-intervention)	1.32	0.76	2.29
Phase 1 (roll-out period)	Phase 0 (pre-intervention)	2.39	1.31	4.36

Table 2 shows documentation of competency in women’s health-related procedures for residents who graduated in 2019, 2020, 2021, and 2022. There was no process for such documentation for the class of 2019. Significant changes in competency documentation occurred over time for the classes of 2020, 2021, and 2022.

**Table 2:** Residents with 15 or more documented women’s health procedures and documented competency in five or more women’s health-related procedures at the time of graduation Pap smears and maternity care procedures are excluded because of the context of this project. Those were addressed by another curriculum and were not a problem at baseline.

Class	Residents with 15 or more documented women’s health-related procedures, N (%)	Residents with documented competency in five or more women’s health-related procedures
2019	3/6 (50%)	Not applicable: no process available at the time of graduation
2020	5/6 (83%)	Process was created, but no documentation was found at the time of review
2021	5/5 (100%)	5/5 (100%)
2022	7/7 (100%)	6/7 (85%)
2023	7/7 (100%)	7/7 (100%)

## Discussion

This study demonstrates that a procedurally oriented, multifaceted bundled intervention was associated with significantly improved training in outpatient women’s health-related procedures among FM residents. This approach could help meet the core procedural competency benchmarks required for accreditation by the ACGME [1] and those suggested by the workgroup from the STFM [2]. The intervention increased the average number of women’s health-related procedures performed, from two per resident per month in the pre-intervention phase to six per resident per month in the post-intervention phase, as shown by Poisson regression analysis and phase-by-phase comparison using X-mR SPC charts. The number of graduates with documented performance and competency in women’s health-related procedures at the time of graduation also increased over time. These findings support the study hypothesis that targeted interventions can enhance residents’ training in outpatient women’s healthcare.

The results of this study are significant because they address a critical training gap in FM residency programs, particularly in underserved areas where access to specialized obstetrics and gynecology services may be limited, and FM physicians often serve as the primary providers of women’s healthcare [5,9]. There have been reports of changes in the scope of practice among FM physicians over time [10,11]. The number of FM physicians providing a broader spectrum of care has been declining [12], including care of children [13], maternity care [14], and other gender-specific healthcare needs for women [15], despite clear associations between comprehensive care provided by FM physicians and the realization of the Triple Aim [16]. The reasons for this shift have also been a topic of debate. Coutinho et al. demonstrated that the gap between intended and actual scope of practice is more likely due to available training opportunities than to generational differences [17]. Our study shows that deliberate and thoughtful changes in training processes are associated with increased opportunities to gain experience in women’s health-related procedures. The results align with previously published literature, which emphasizes the importance of dedicated procedural training in improving physician confidence and procedural volume [3,6]. Studies show that structured interventions, such as simulation training and dedicated procedure clinics, significantly enhance procedural competency among residents. Our findings corroborate these outcomes, particularly highlighting the role of a dedicated women’s health-related procedure clinic as a critical factor. However, this study adds to the existing literature by demonstrating the effectiveness of a bundled intervention that includes preceptor training and resident development through didactics and simulations, alongside the establishment of a dedicated procedure clinic.

Despite its strengths, the study has limitations. The COVID-19 pandemic disrupted implementation during phase 2, potentially influencing outcomes. Additionally, the single-institution setting limits generalizability, and long-term retention of procedural skills and their impact on patient outcomes were not assessed.

## Conclusions

This study demonstrates that the implementation of a multifaceted, bundled intervention was associated with improvements in residents’ training in women’s health-related procedures. It highlights the potential of such an intervention to address training gaps in FM residency programs. Future research should focus on replicating this model across diverse settings and evaluating its sustainability and broader impact on healthcare delivery.
